# Patients’ perception of types of errors in palliative care – results from a qualitative interview study

**DOI:** 10.1186/s12904-016-0141-4

**Published:** 2016-08-11

**Authors:** Isabel Kiesewetter, Christian Schulz, Claudia Bausewein, Rita Fountain, Andrea Schmitz

**Affiliations:** 1 Ludwig-Maximilians-University, Department of Anaesthesiology, Munich University Hospital, Munich, Germany; 2 Ludwig-Maximilians-University, Department of Palliative Medicine, Munich University Hospital, Munich, Germany; 3 Maudsley Training Programme, Institute of Psychiatry, Psychology, and Neuroscience, King’s College, London, UK; 4 Department of Psychosocial Oncology and Palliative Care, Dana-Farber Cancer Institute, Boston, USA; 5 Interdisciplinary Center for Palliative Medicine, Heinrich Heine University Hospital Düsseldorf, Düsseldorf, Germany; 6 LVR Clinic of Psychiatry, Psychosomatic and Psychotherapy for children and adolescence, Viersen, Germany

**Keywords:** Error, Medical error, Palliative care, Patient’s perspective

## Abstract

**Background:**

Medical errors have been recognized as a relevant public health concern and research efforts to improve patient safety have increased. In palliative care, however, studies on errors are rare and mainly focus on quantitative measures. We aimed to explore how palliative care patients perceive and think about errors in palliative care and to generate an understanding of patients’ perception of errors in that specialty.

**Methods:**

A semistructured qualitative interview study was conducted with patients who had received at least 1 week of palliative care in an inpatient or outpatient setting. All interviews were transcribed verbatim and analysed according to qualitative content analysis.

**Results:**

Twelve patients from two centers were interviewed (7 women, median age 63.5 years, range 22–90 years). Eleven patients suffered from a malignancy. Days in palliative care ranged from 10 to 180 days (median 28 days). 96 categories emerged which were summed up under 11 umbrella terms *definition, difference, type, cause, consequence, meaning, recognition, handling, prevention, person causing* and *affected person*. A deductive model was developed assigning umbrella terms to error-theory-based factor levels (definition, type and process-related factors). 23 categories for *type of error* were identified, including 12 categories that can be considered as palliative care specific. On the level of process-related factors 3 palliative care specific categories emerged (*recognition, meaning* and *consequence of errors*).

**Conclusion:**

From the patients’ perspective, there are some aspects of errors that could be considered as specific to palliative care. As the results of our study suggest, these palliative care-specific aspects seem to be very important from the patients’ point of view and should receive further investigation. Moreover, the findings of this study can serve as a guide to further assess single aspects or categories of errors in palliative care in future research.

## Background

In recent years patient safety and medical errors have become increasingly important topics in the general public as well as in research and health policy. Besides numerous reports in the media [1], establishment of institutions such as the Patient Safety and Quality of Care Work Group of the European Commission [2] and a growing number of scientific articles in diverse medical subspecialties underline this development. As it is well established that errors in health care contribute substantially to patient distress, morbidity and mortality [3–5], many medical disciplines have developed initiatives for detection and prevention of errors [6, 7]. Moreover besides the obviously error prone disciplines such as surgery or anaesthesiology also “smaller” medical specialities focused more on medical errors, for example geriatrics considering polypharmacy and medication errors in older patients [8, 9].

Palliative care as a “small” discipline with its own and special issues, such as the acceptance of death, will need to take that challenge in the future. As a recent literature review showed, there is little empirical research on errors in palliative care to date [10]. Patients in palliative and end-of-life care are more likely to be particularly vulnerable to medical errors. Even if no systematic data on the frequencies of errors in palliative care can be provided at present, a retrospective cohort study on an inpatient specialist palliative care service found that 62 % of patients suffered from symptomatic adverse events [11]. Moreover, a survey of professionals (physicians, nurses, psychologists, social workers, spiritual care workers and other professionals) on errors in palliative care showed that palliative care provides a number of special aspects like the multi-professional team approach, patient and family as unit of care, the importance of communication, and the acceptance of death that may additionally warrant an investigation on errors in this specialty [12].

Knowledge about errors in palliative care is limited and the few existing studies include the perspective of medical professionals only. Patients’ needs differ from those identified by health care professionals [13, 14], but until now studies about patients’ perceptions of errors in palliative care are not available. For a holistic understanding of errors in palliative care and for future approaches how to handle patient safety in this medical field, the evaluation of the perspectives of patients – those who are the focus of care - is needed.

The aim of this study was to explore how palliative care patients perceive and what they think about errors in palliative care in order to establish an understanding of what an error in palliative care is and which meaning it has for the patients. As secondary objectives, the study wanted to detect areas, causes and consequences of errors in palliative care.

## Methods

In the following, the consolidated criteria for reporting qualitative research (COREQ) checklist for reporting qualitative studies was adopted for the presentation of our data [15].

### Theoretical framework

The study is based on the theoretical framework of qualitative content analysis after Mayring, which has been used in palliative care research before [16–19]. Interviews were problem-centered combining communication strategies as primary approach to the problem and imaginative and semi-structured prompts to stimulate narratives in a secondary step [20].

### Participant selection

Patients who met the following inclusion criteria were recruited: incurable advanced disease (malignant: all kinds of cancer; non-malignant: chronic organ failure, neurological diseases, HIV/Aids) and experience of at least 1 week under specialist palliative care prior to the interview.

To ensure a wide variation of patients’ perspectives of errors in palliative care, a purposive sampling approach and heterogeneous sample was chosen [21] and participants were selected according to a pre-defined sampling frame that included patients’ age, sex, diagnosis and the timeframe the patients had received palliative care.

Participants were approached via palliative care units, palliative care hospital consultation teams or the palliative home care team at the two participating German university hospitals.

### Setting

All interviews that took place in the hospital were recorded in a private setting. In three of four interviews conducted at the patients’ home one relative was present during the interview but did not participate.

### Research team and reflexivity

Interviews were conducted by two female physicians (IK and AS). To account for possible physician/interviewer role-conflict, interviewers were not in charge of the medical care of those interviewed in both institutions. IK introduced herself as a physician in anaesthesiology and palliative care working as a researcher in the Department of Palliative Medicine at the Munich University Hospital. AS introduced herself as the medical chief and a researcher of the Interdisciplinary Center for Palliative Care at the Dusseldorf University Hospital.

### Data collection

A topic guide was developed based on theoretical knowledge, prior research and group discussions within the research team. The topic guide covered the general understanding of an error, errors in palliative care and their characteristics and consequences of errors in palliative care (see Appendix 1).

In addition to the interviews, further information such as demographic data, diagnosis and co-morbidities were collected from the patients’ medical charts. All interviews were tape-recorded and transcribed verbatim.

Data saturation was discussed within the research team and after 12 interviews both interviewers agreed that saturation of data was reached.

### Data analysis

All interviews were analysed according to the coding paradigm steps of qualitative content analysis [22].

Two researchers (IK and AS) paraphrased the text independently and emerging discrepancies were resolved by communicative validation. In a consecutive group discussion with a third researcher (CS) all paraphrases with disagreement were discussed and a consistent level of abstraction was agreed on.

In a subsequent multi-level process, a coding scheme was developed and all paraphrases were added to their respective categories.

To improve trustworthiness of the coding intercoder-reliability was measured for three interviews (25 % of the material). The overall concordance for all three interviews was 73.5 % (*n* = 181 arguments, 48 differences). Intercoder-reliability increased over the rating process from interview 1 with 71.9 %, to interview 2 with 73.5 % to interview 3 with 76.2 concordance.

Interviews were analysed using Microsoft Word and Excel (Microsoft Office 2011).

## Results

Twelve interviews were conducted between September 2013 and January 2014. Overall, 18 patients gave consent to participate. Six patients could not be interviewed because of increasing weakness of the patient (*n* = 2), deterioration of their general condition (*n* = 2), cognitive impairment (*n* = 1) and refusal of the patient’s family (*n* = 1).

Seven interviews were conducted in Munich, the other five in Düsseldorf. Seven of the interviewed patients were women, the median age was 63.5 years (range 22 to 90 years). Eleven patients suffered from advanced cancer. Patients were a median of 28 days in specialized palliative care (range 10 to 180 days). The median interview time was 39 min (range 18 to 59 min). Table 1 shows the characteristics of the sample in more detail.

**Table 1:** Characteristics of interviewed participants

Interview number	Participant number	Sex	Main diagnosis	Other diagnoses/symptoms	Days in palliative care	Setting of the interview	Approach via
1	MUC 2	f	ovarian cancer	metastasis of the bone, lung, gut and peritoneum; spinal cord compression with paraplegia, pain	12	palliative care unit	palliative care unit
2	MUC 3	m	colon cancer	metastasis of the liver and peritoneum, pain, sleep disorder	10	home	outpatient palliative home care service
3	MUC 4	m	chronic obstructive pulmonary disease (COPD)	heart failure, diabetes, osteochondrosis, dyspnoea	180	home	outpatient palliative home care service
4	MUC 6	f	glioblastoma	epilepsy, difficult social conditions	45	home	outpatient palliative home care service
5	MUC 7	m	pancreatic cancer	pain, cachexia, nausea	30	home	outpatient palliative home care service
6	MUC 8	f	lung cancer	dyspnoea, pain	12	palliative care unit	palliative care unit
7	MUC 10	f	leiomyosarcoma	pain, provision of care unclear	23	palliative care unit	palliative care unit
8	DUS 1	f	anal cancer	local infiltration, pain, hypercalcemia, oral thrush, anxiety, sleep disorder	28	palliative care unit	palliative care unit
9	DUS 2	f	breast cancer	metastasis of the bone; instable fractures of the spine; pain, anxiety	40	palliative care unit	inpatient palliative care consultation service
10	DUS 5	m	cancer of unknown primary (CUP)	metastasis of the bone, lung and adrenal gland, pain oesophageal thrush, cachexia, obstipation, immobility, nausea, hypercalcemia, anxiety, restlessness	28	palliative care unit	palliative care unit
11	DUS 7	f	glioblastoma	nausea, pain, vertigo, sight disorder	53	palliative care unit	palliative care unit
12	DUS 8	m	bladder cancer	metastasis of the liver, local infiltration, depression, pain, nausea, immobility	10	urological ward	inpatient palliative care consultation service

### Categories

During the inductive coding process 96 categories emerged from the original data that were subsumed to the following 11 umbrella terms:

- Definition
- Differences
- Type
- Causes
- Consequences
- Meaning
- Recognition
- Handling
- Prevention
- Person causing
- Affected person

### Three level model of patients’ perception of errors in palliative care

**Fig. 1:**
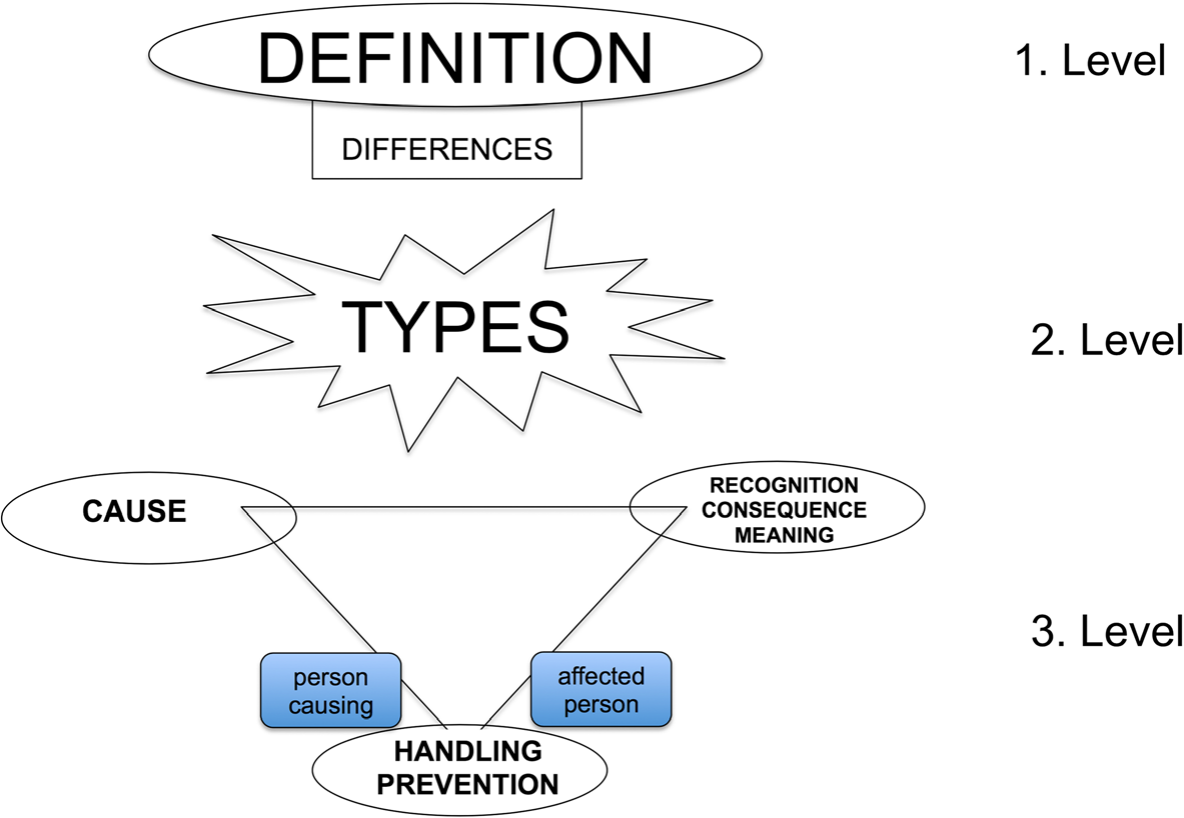
Three level model of patients’ perception of errors in palliative care integrating the eleven umbrella terms

In a final deductive step, all umbrella terms were classified in a system of three thematic levels (see Fig. 1): on the first level is, general themes considering definition of and differences between errors in general, errors in medicine and errors in palliative care were grouped together. On the second level is a grouping of types of errors and the third level contained action-related categories (causes of errors; consequences, recognition, and meaning of errors; handling and prevention of errors). All 96 categories, subsumed to umbrella-terms and levels are presented in Appendix 2.

As umbrella terms and categories cover a very large range of different items and issues, a holistic examination of all of them would go beyond the scope of this paper. Therefore in this paper, we focus on reporting findings for “types of error” (second level) and “recognition”, “consequence” and “meaning” (third level).

### Types of error

Under the umbrella term “types of error” 23 categories were subsumed, of which 10 categories applied to types of errors that exist both in medicine and in palliative care and 13 types of errors that were described specifically for palliative care. Table 2 shows an overview of all types of errors that may be considered specific for palliative care with exemplifying citations.

**Table 2:** Overview of all categories of *type of error* with exemplifying citations that may be considered as specific for palliative care

Breach of the patient’s wishes or patients advanced directives	“An error in PC would be a deviation … from the agreed rules of the game.” (DUS 5)
	“Well, I myself have an advance directive and I expect that what is written there will be followed. …I wrote it with my full mental capacity, I gave it a lot of thought. (If the doctor does not follow the advance directive) that would be an injury to me personally. Because he in fact ignores what I have said, he actually ignores my opinion…that is like a law, like other directives, if you submit yourself to these directives and state that you will work under these criteria then you can’t do anything because it is set this way, you can’t bully and not rebel against it, nothing helps.” (DUS1)
Trust/empathy	“The patient should feel safe with those people and should have trust in them, […] then they let themselves be helped differently. And when that is not the case, that would be a big mistake.” (DUS2)
	“The purpose of palliative care is that patients do not get conventional medical treatment, […] where everything is measured, checked, tested and examined but that a doctor takes his time and turns to the patient and approaches the patient, perhaps to talk about spiritually, psychologically supporting the patient in his position where he may not have long to live […] If there […] is no space to express feelings and actually establish good communication with the patient, that I think would be a mistake for me, if a doctor does not bring this ability along.” (MUC7)
Psychologically	“In the psychological area very bad mistakes can happen […] there are conversations about people, for example, as if they were not present, that some people might be treated rough, […] in this context people could be seriously injured, psychologically damaged and that is a very big mistake especially in this area of care to leave people alone, in their pain, in their fears.” (DUS1)
	(A psychological error would be) “when one might not recognize what the patient really needs psychologically, communicative, when one can’t respond to his fears or inspire confidence or can really provide consolation to him in the last hours.” (MUC7)
Communication	“It is important for the patient to be spoken to … yes even if it’s just a homeless person. You have to talk with them also. One talks with a dog so why should you not do it with a person. You have to try that even if they are stubborn, the people …, one has to talk to people.“(MUC3)
Information	“I think, a mistake might be, if you are not honest with people. I would view this as a large mistake, because I believe, that it is very important for the patients, … and many won’t do it, not because they are mean, but because they think it will calm us … but I believe that is not the issue, when you are ready, then you also want to know the truth.” (DUS2)
Resuscitation	“They are getting the signature that there will not be any resuscitation efforts. I would view that as a mistake, if there would be resuscitation efforts in this case, because it was noted in the palliative care ward and when someone is doing that it is maybe twice as difficult because I am not in a regular hospital or … in a regular emergency room, but I am in a hospital where I expect that people work alongside this dying process and not work against it.” (DUS1)
Prognosis	“A mistake in palliative medicine would be to state, for example … so now it’s time to head out to hospice and then one notices after three months, oops I am still here.” (MUC1)
Nursing measures	“In the nursing area there could be mistakes, that some type of lotions were incorrectly used.” (DUS1)
Insufficient symptom management	“… basically I can only hope… that I can depart this world without having pain. And that is what I demand and I would think it a big, big mistake if that would not be the case.” (MUC1)
Preparation for death not possible	“It would be a mistake if in PC the patient would not get the opportunity to prepare himself for death, which is only offered sparingly by society… so that they will give assistance in this area, towards this topic, in a serious conversation to get used to this and not just by saying, now you have to turn to the right side or left side and then the pulmonary therapist will come and tickle your foot.” (MUC2)
Involvement of relatives	“A mistake would have been from my point of view, if they (palliative care ward) would not include the relatives.” (DUS2)
Sedation	(A mistake in the palliative medicine would be) “if one would just be sedated… when from there on nothing else would work anymore.” (MUC6)
Patient as guinea pig	“To use patients for experiments, that I find is a mistake. … if everyone is trying something, another little bit here and there, such guinea-pig-like, that would be a mistake … maybe they can help someone at a later point … but for the patient himself I would think this a mistake, if they try things on me to maybe help others, when one is still here.” (DUS2)

### Affected person, consequence, meaning and recognition

As categories for “affected person”, the three categories *patients, professionals* and *others emerged*.

The umbrella term of “recognition” has only one category. The category *patient* included all statements that reflected on a potential recognition of errors by the patient:

“… in such a palliative care situation, it can be that pain management is not properly adjusted. And you are unaware of this and you think, because you trust them, that this is the best you can get…so you are not aware that this is an error.“(DUS5)

The umbrella term “consequence” covered nine categories.

Five of those categories considered the patient as affected person: *shortening of life, mental burden, distressing symptoms, physician-patient-family-relationship strain* and *limitation of mobility/autonomy*.

*Shortening of life, strain of the patient-physician relationship* and *mental burden* as consequence of an error were described by one patient as follows:

“… the worst is that someone has died (by an error). It can also happen that the patient has totally lost all trust. […] and as a consequence you probably don’t’ want to participate in anything or don’t want to do anything or are afraid to do anything.”(DUS2)

The category *limitation of mobility/autonomy* can be found in the answer of one patient to the question what an occurred error means for palliative care patients:

“I always think the head is the most important part to function in a person . Whether I can walk now or not, there is a wheelchair. Whether I can properly go to the bathroom right now or not, there’s medication for that. But that the head functions properly, I think that is tremendously important. That you can still think for yourself a bit. … When one is writhing in pain, the head can’t think cleverly anymore.“(MUC6)

Considering consequences for the professional, the three categories *judiciary, physician’s reputation* and *disciplinary actions* emerged.

The single category *compensation* did not consider consequences for a certain person but included statements in which patients described their belief that many errors remain without consequences or that consequences are not compensated.

The umbrella term “meaning” has five categories: *exceptional circumstances of the patient*, *appraisal in the context of approaching death, death caused by an error – terrible, death caused by an error – less terrible* and *individually different*.

Several patients described a special meaning of errors in palliative care based on the exceptional circumstances of the patient in a palliative care situation a different *appraisal in the context of approaching death*:

”… an error… would be really horrible for that person, since he is so dependent and can’t wiggle out of the situation, in that moment he is really dependent on people and expects that those people do their work nearly 100% perfect, and really try to avoid any mistakes. Because otherwise we would not be here [in palliative care], we could lay at home and close our eyes” (DUS1)

“… you are in an exceptional situation, if you are under palliative care. One is immediately in another world and everything you encounter is valued differently. Everything is so totally related to my life, my end of life, my very limited time.... Things that are just mentioned (by the palliative care physicians), may be received by patients in a totally wrong light,“(MUC5)

Considering the possibility of death caused by an error at the end of life or in a palliative care situation, some patients described the meaning of such an event as *terrible* and some patients considered it as *less terrible* or even helpful:

“Because I actually would like to live for 10 more years … if the time would now be limited through an error by others, I would be a little bit annoyed. Because … when I wake up in the morning, then I think to myself: beautiful, yesterday was such a beautiful sunset, sunrise today, super, managed another day.“(MUC6)

“I think nowadays sometime helpful errors happen … I am just thinking about a patient who might suffer and can’t die, and they give him by mistake a few too many sleeping pills and a little too much morphine and then he does finally sleep.“(DUS4)

## Discussion

This study explored the perceptions and thoughts of palliative care patients about errors in palliative care to generate an understanding of such and to detect areas, causes and consequences of errors in palliative care. To the best of our knowledge, this is the first study that explores errors in palliative care from the patients’ perspective. Errors specific to palliative care were identified on the levels of definition, type of error and process-related factors.

Identified types of errors in palliative care are comparable with types of errors in medicine and those identified by palliative care professionals. Especially diagnosis and treatment errors or medication errors are frequent and well acknowledged types of errors in medicine [23, 24] and they have also been noticed in the palliative care environment [10, 12]. Nevertheless, a direct comparability or even transferability of approaches for detection, handling and prevention of errors is not that easy. On medication errors, for example, much research is performed and concepts are developed to reduce errors in drug prescriptions and drug administration in health care [25, 26] but the transferability of those concepts to a palliative care population may be limited. Due to polypharmacy being common, high degrees of comorbidities and limited life expectancies in palliative care patients, different concepts will be required to reduce medication errors and to enhance patient safety and drug related quality of life [27]. Many errors, especially with regard to medication errors might not be noticed or realised by the patients and those errors they do know about might often be less consequential to their survival.

Some types of palliative care specific errors, for example, lack of adequate symptom control or resuscitating a patient with DNR order, are viewed as the paradigm differences between curative medicine and palliative care. As the relief and prevention of symptoms in terms of a best possible symptom control and quality of life instead of maximum prolongation of life are the main fundamentals and goals of palliative care, not achieving these goals may be considered a failure or error [28]. Those “common” types of errors in palliative care are addressed by current research projects in palliative care, as all of them intend to reduce these errors, be it by improvements in drug therapy or melioration of provision of care or through education of medical staff or guidance in ethically challenging cases – basically by improving the level of palliative care itself.

The results of our study suggest that communication errors and errors in professionalism play a central role in patients’ perception of errors in palliative care. Future research should include less obvious types of errors in palliative care, errors that in our study appeared in the categories *communication, information* or *trust/empathy*. A study by van Mook et al. in the Netherlands revealed that insufficient clarification or unclear information, disrespectful communication and lack of sympathy and empathy were three of the six most frequent reasons for healthcare complaints from patients or families [29]. This study included data from a general hospital and showed that from the patients’ perspective professionalism and professional behaviour are highly important but undervalued by health care providers and professionals. In palliative care, where the patient-professional interaction is a core element of daily practice, special attention should be paid to those aspects of professionalism and related types of errors that were also described by the patients in our study.

The categories *information*, *communication, breach of the patient’s wishes or patient’s advance care directives* and *not perceived as an informed patient* touch the topics of autonomy. Autonomy at the end of life, especially considering decision-making is controversially discussed [30]. A large number of palliative care physicians in the world do not inform their patients about the terminal stage of their illness [31]. Palliative Care patients identified knowing what to expect about their physical condition as a major need when they were asked about preferences regarding end of life preparations [32]. Supporting these findings, the lack of honest information about physical condition and prognosis as well as shared decision-making was described as an error in palliative care in our interviews.

In our study different categories regarding meaning of errors in palliative care emerged: *exceptional circumstances of the patient, appraisal in the context of approaching death, death caused by an error – terrible, death caused by an error – less terrible* and *individually different*. The few publications on meaning of an error in medicine other than palliative care focus on the meaning of living with preventable medical harm from the participants’ perspective, the impact of communication and relationship, trust and patient-centeredness [33]. Although these issues did not emerge in our study in the context of meaning of an error, this should not be misinterpreted as not being important in palliative care. However, our findings suggest that the topic of meaning of an error in palliative care needs to be studied in more detail and the relationship of types of errors and the meaning for the patient should be explored. Those subsequent studies should aim to generate a better understanding of the factors that influence patients’ appraisal of severe or slight, important or unimportant, restrictive or non-restrictive meaning and also how death caused by an error is judged in a palliative care situation.

In summary, from the patients’ perspective several categories especially for type and meaning of errors imply that they are specific for palliative care and that they could be overlooked from the professionals’ perspective. This study was the first to open the field into this area of research and produced a wide range of findings that have to be explored in more depth in future studies.

### Limitations

This study has clear limitations. An indispensable aspect that has to be discussed in the context of limitations of qualitative studies is generalization [21]. For the findings of our study we cannot claim an overall generalisation, but we took precautions to enhance validity and reliability and thus the strength of our results. Nevertheless, a number of limitations considering reliability and validity can be found.

Moreover, this study is based on the experience of 12 patients from two different centers in Germany who received palliative care in their respective centers only. To scrutinize the relatively large number of categories found in this qualitative study, future research should include more patients and palliative care centers and palliative care experiences from diverse health care settings.

The data presented is limited by the degree of awareness and understanding of medical error by the participants. Patients might not be aware of many unnoticed errors happening every day – for example medication errors that are known and prone to happen often and to stay unnoticed - and that the patients’ general understanding of an error may be very ambiguous [34, 35].

Considering the methodology of the present study no participant review was used in terms of returning transcripts and findings to the patients to confirm or comment on them. This step may enhance the quality of the findings and may give more rigor to it [36].

## Conclusion

In times where more and more studies are conducted to generate “hard facts” and high-grade evidence to develop standards that define right and wrong, we consider the present study as an important counterbalance to this development through the integration of the patients’ perspective, especially in the area of medical error management.

Errors in palliative care touch similar aspects as in other areas of medicine but there are also aspects specific to palliative care mainly related to issues such as communication, professionalism or advance care planning.

The issue of errors in palliative care and particularly errors from the patients’ perspective needs much more clinical and scientific engagement and the recent study may be seen as a baseline and index of important aspects.

Therefore, the three level model developed in the present study including 1. definition of and differences between errors, 2. types of errors and 3. causes, consequences, recognition, meaning, handling and prevention of errors, gives a specification of issues to explore in more depth and detail in future projects.

## Abbreviations

AS, Andrea Schmitz; CB, Claudia Bausewein; CS, Christian Schulz; DUS, Dusseldorf; IK, Isabel Kiesewetter; MUC, Munich

## Appendix 1: Topic guide

Topic guide for in-depth interviews with patients for the study: “Patients’ perception of errors in palliative care – a qualitative interview study

Research objectives

- to explore how palliative care patients perceive and what they think about errors in palliative care and to generate an understanding of what an error in palliative care is and which meaning it has for the patients.

The following is a topic guide to prompt patients in the interview in different directions regarding errors in palliative care. It is not intended to ask every of the following questions word by word but to have the topic guide as an aid memoire whether all important areas are covered.

## Start (interviewer)

Introduction of the interviewer, the study, and its purpose

Estimated duration of the interview

Consent and confidentiality: written consent, tape recording, what will happen with the data

Possibility to stop at any time or decline any questions without any consequences

## Background

Patients background, current medical and psychosocial situation, care situation etc.

## Topics

1. Introduction
  - How would you describe an error in general?
  - What would you call an error in medicine?
2. Errors in PC
  - What do you call an error in palliative care?
  - Can you describe an example of an error in palliative care?
  - Have you ever experienced yourself an error or something that you would call an error in your palliative care treatment?
3. Characteristics of errors in PC
  - What different types or areas of errors in palliative care can you imagine?
  - Which people can commit an error in PC?
  - Which people can be affected by an error in PC?
  - What do you consider as main causes of errors in palliative care (situation, settings, factors…)?
4. Consequences of an error in PC
  - What do you consider as main consequences of errors in palliative care?
  - How should errors in PC be managed? (coping and compensation strategies, communication, what is important, acknowledgment or not and by whom)
  - How could errors in PC be prevented?
  - How do you evaluate the impact or meaning of errors in palliative care/at the end of life? (burden; emotional reaction, lost of trust or hope, fear, feeling helpless…)
  - What can you tell me about the meaning of guilt at the end-of-life?
5. Debriefing
  - Is there anything else you want to say or talk about? Is there anything we did not cover?
  - How did you find the interview?
  - Any questions uplifting or upsetting?
  - Was the length of the interview okay for you?
  - Any questions which were helpful or interesting?
  - Do you have further comments?

## Appendix 2

**Table 3:** Table of all 96 categories, subsumed to umbrella-terms and levels

Level	Umbrella term	Category	Classification
1	Definition (*n* = 21)	Interpersonal subjectivity Intrapersonal subjectivity To err is human Wrong reaction Wrong decision Wrong assessment Deviation from a plan Harm Wrong outcome Difficult	comprehensive
		Error of a person Doing nothing	exclusive: general error
		Violation of learned facts Deviation from a standard Need for progress Not maximizing the best for a patient	exclusive: medical error
		Patients personal opinion Dignity Consideration of the physician Acceptance of symptom management without diagnosis Difficult	exclusive: error in palliative care
	Differences (*n* = 4)	Weight Standards	general error/medical error
		Existing differences No differences	medical error/error in palliative care
2	Types (*n* = 23)	Diagnosis Therapy Missed alternative treatment options Medication administration (wrong administartion or mix-up) Medication dosage Medication - useless or not meaningful Depersonalization Not perceived as an informed patient Help not fast enough Confidentiality	not palliative care specific
		Breach of the patient’s wishes or patient’s advance care directives Trust/empathy Psychologically Communication Information Resuscitation Prognosis Nursing measures Insufficient symptom management Preparation for death not possible Involvement of relatives Sedation Patient as guinea pig	palliative care specific
3	Causing person (*n* = 6)	Physicians Nurses Carers in general Patients Relatives Others	
	Causes (*n* = 20)	Uninfluenceable Character traits Environmental factors Chain reaction	causes for errors in general
		Rigidity Ambition Paternalism Infallibility of the physician Acceptance of errors due to imminent death	related to the physician
		Lack of time Lack of personnel Teamwork Communication Coordination Antipathy Training/knowledge Human weaknesses Lack of evidence Inadequate care motivation	related to the nurses/carers
		Lack of patient resources	related to the patient
	Affected person (*n* = 3)	Patients Professionals Others	
	Consequences (n = 9)	Shortening of life Mental burden Distressing symptoms Physician-patient-family-relationship strain Limitation of mobility/autonomy	affected person: patient
		Judiciary Physician’s reputation Disciplinary actions	affected person: physician
		Compensation	no certain affected person
	Meaning (*n* = 5)	Exceptional circumstances of the patient Eppraisal in the context of approaching death Death caused by an error - terrible Death caused by an error - less terrible Individually different	
	Recogintion (*n* = 1)	Recognition of errors by the patient	
	Handling (*n* = 9)	Culture of errors Public disclosure Disclosure to the team Disclosure to the patient Reparation Apology Learning from errors Discussion Cause analysis	
	Prevention (*n* = 10)	Teamwork Communication Information Advanced directives Education/knowledge Work routine Staff Time Patient collaboration Patients’ resources	
